# Reversing oncogenic transformation with iron chelation

**DOI:** 10.18632/oncotarget.27866

**Published:** 2021-01-19

**Authors:** Gina Abdelaal, Stephany Veuger

**Affiliations:** ^1^Department of Applied Sciences, Faculty of Health and Life Sciences, Northumbria University, Newcastle upon Tyne, UK

**Keywords:** iron chelator, oncogenesis, selective cytotoxicity, hallmarks of cancer, NDRG1

## Abstract

Cancer cells accumulate iron to supplement their aberrant growth and metabolism. Depleting cells of iron by iron chelators has been shown to be selectively cytotoxic to cancer cells *in vitro* and *in vivo*. Iron chelators are effective at combating a range of cancers including those which are difficult to treat such as androgen insensitive prostate cancer and cancer stem cells. This review will evaluate the impact of iron chelation on cancer cell survival and the underlying mechanisms of action. A plethora of studies have shown iron chelators can reverse some of the major hallmarks and enabling characteristics of cancer. Iron chelators inhibit signalling pathways that drive proliferation, migration and metastasis as well as return tumour suppressive signalling. In addition to this, iron chelators stimulate apoptotic and ER stress signalling pathways inducing cell death even in cells lacking a functional p53 gene. Iron chelators can sensitise cancer cells to PARP inhibitors through mimicking BRCAness; a feature of cancers trademark genomic instability. Iron chelators target cancer cell metabolism, attenuating oxidative phosphorylation and glycolysis. Moreover, iron chelators may reverse the major characteristics of oncogenic transformation. Iron chelation therefore represent a promising selective mode of cancer therapy.

## INTRODUCTION

Iron is vital for normal cell growth and survival. Cancer is an evolutionary maverick, which exploits its trademark genomic instability to drain environmental resources. As an enzyme cofactor, iron is responsible for many cellular processes including mitochondrial metabolism and DNA synthesis [1–3]. As iron can drive cellular proliferation, cancer cells have an adapted iron metabolism allowing increased iron accumulation. Studies have demonstrated cancer cells have an aberrant expression of iron metabolism genes, as well as an overexpression of iron import proteins and underexpression of iron export proteins [4–7]. This has led to iron accumulation being considered as a target for cancer therapies. Among the potential therapies which target iron metabolism, iron chelators are one of the most well studied. Iron chelators selectively deplete cancer cells of iron, exploiting cancer’s iron addiction – a trait displayed by a range of different cancers.

Deferoxamine (DFO) was the first iron chelator taken forward for clinical trials in 1987. Initially DFO was designed as a treatment for iron overload, but promising research conducted in cell models prompted clinical testing [8–11]. Of note, in a Phase II trial neuroblastoma patients treated with DFO displayed reduced bone marrow infiltration and one patient had a significant decrease in tumour mass [12]. Overall patients’ response to DFO has been variable with some patients showing complete or partial response and some patients showing no response (See Supplementary Table 2) [12–16]. This has been attributed to the poor lipophilicity of DFO, as well as its rapid clearance by the kidneys and poor absorption in the small intestine [17, 18]. Moreover, DFO is administered through continuous infusions, which is inconvenient, time-consuming and painful for patients [19]. This mixed bag of results has fuelled further functional and structural studies aimed at designing improved iron chelators as potential anticancer therapies.

The thiosemicarbazone class is a later stage of iron chelator evolution which manifested in 1992 [20]. Unlike their predecessor DFO, thiosemicarbazone chelators are capable of inducing reactive oxygen species (ROS). Triapine (3-AP) is a thiosemicarbazone; its primary mode of action is thought to be ribonucleotide reductase inhibition with a higher potency than commonly used ribonucleotide reductase inhibitor, hydroxyurea [21, 22]. Interestingly, tumours resistant to hydroxyurea retain sensitivity to triapine [23]. Triapine was taken forward to clinical trials in 2002 where it was successful with blood cancers, but not solid tumours (See Supplementary Table 2) [24–27]. Triapine acted as a radiosensitiser during cervical, vaginal and ovarian cancer clinical trials (See Supplementary Table 2) [28, 29]. Another issue that manifested was a short-lived patient response, suggesting patients develop resistance [24, 30]. Triapine is rapidly metabolised and its metabolite is inactive, rendering it ineffective against solid tumours [31, 32].

More structural and functional studies lead to the emergence of Dp44mT – a terminally demethylated triapine derivative. ROS induction is pivotal for Dp44mT function as antioxidants inhibit Dp44mT cytotoxicity [33]. Dp44mT is almost 50-fold more potent than its predecessor triapine. Dp44mT is effective *in vivo*; treated mice experience no changes in body or organ weight and little change in hematologic indices [34, 35]. New innovative ways of drug delivery have been explored in an effort to lower the risk of Dp44mT side effects such as methemoglobin in clinic. Nanoparticles have been used to counteract Dp44mT high cytotoxicity and low bioavailability. This approach is predicted to protect healthy tissues from the cytotoxic effects as the timing and place of the drug release can be controlled [36]. Encapsulating Dp44mT in PLGA nanoparticles enhanced its ability to induce apoptosis and improved its selectivity towards cancer cells [37].

At present many more classes of iron chelators are being taken into consideration as potential cancer therapy candidates. VLX600 is a novel iron chelator capable of targeting both senescent and proliferative cells [38]. VLX600 has been taken forward to a phase I clinical trial where it showed limited adverse effects (See Supplementary Table 2) [39]. Promising preclinical and clinical work may prompt further VLX600 studies. Furthermore, there are many natural compounds, which chelate iron. Natural iron chelators have shown a similar impact on oncogenic signalling pathways as well-characterized iron chelators DFO and Dp44mT (See Supplementary Table 1). Silibinin (isolated from milk thistle), quercetin (plant flavonoid), epigallocatechin gallate (the most abundant component of green tea) have been suggested to have chemo-preventative properties in a range of cancers suggesting potential benefit to their dietary intake, however not without limitations [40–42]. Epigallocatechin gallate has poor bioavailability as it is degraded by the gut microbiota [43]. There are some potential risks associated with quercetin; many animal studies indicated nephrotoxicity, limiting the use of quercetin in patients with pre-existing kidney damage [44]. Although silibinin has been proven safe at high doses its use in clinic still limited by its poor bioavailability, poor solubility in water, and poor absorption in the small intestine [45, 46].

**Figure 1:**
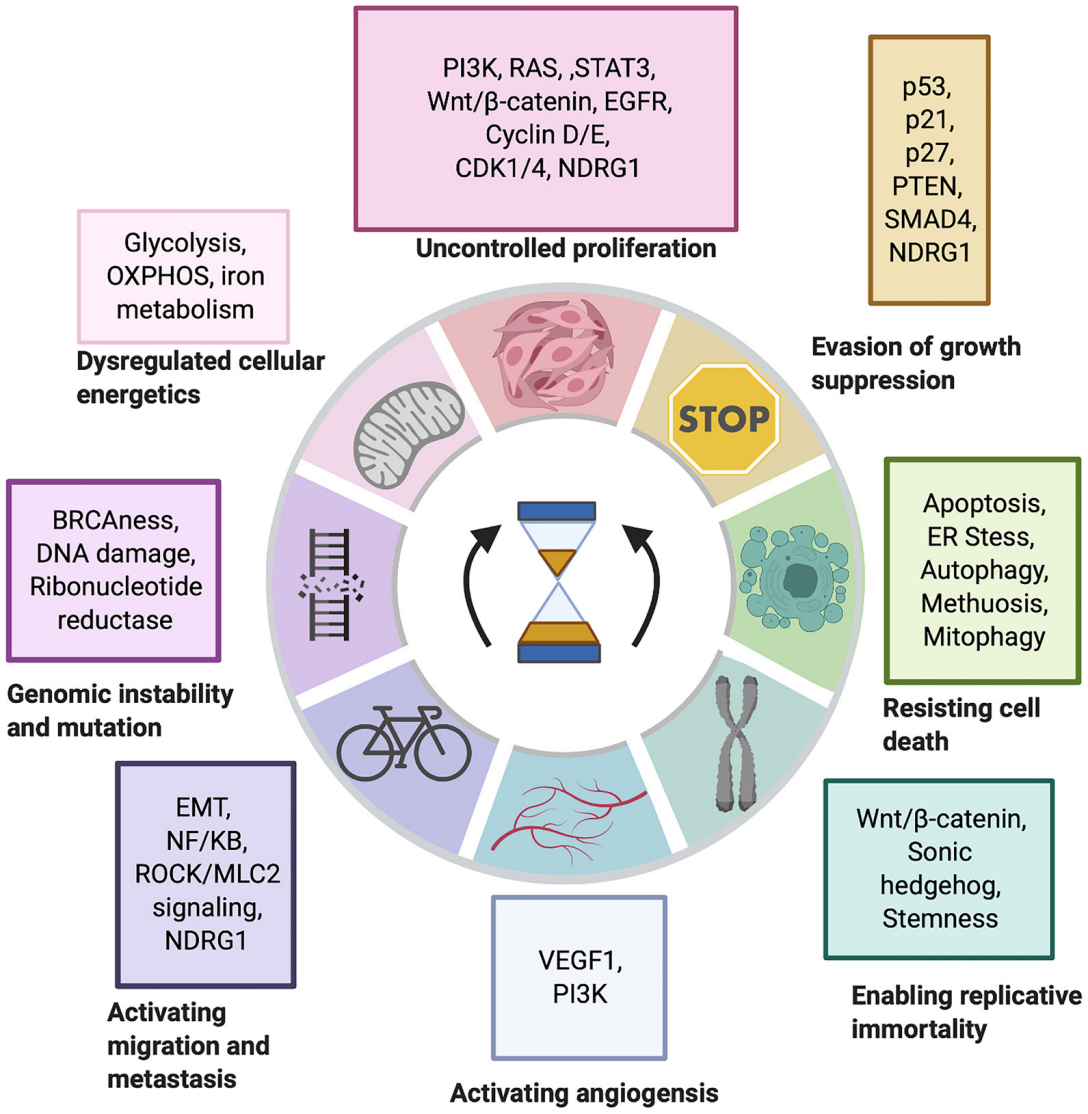
The impact of iron chelators on the hallmarks of cancer. Iron chelators have been shown to reverse many oncogenic signalling pathways associated with each hallmark of cancer with NDRG1 being a common thread. Generated through BioRender.com [47, 48].

This review aims to explore the underlying mechanisms of action behind iron chelator driven cytotoxicity in the context of the hallmarks of cancer established by Hanahan and Weinberg [47, 48] (see Figure 1, Supplementary Table 1). This will in turn support further research into iron chelators as a potential effective anti-cancer therapy.

### NDRG1: The proposed target of iron chelators

N-MYC downregulated gene 1 (NDRG1) plays a critical role in inducing iron chelator mediated cytotoxicity [49]. The NDRG family is composed of 4 members: NDRG1, NDRG2, NDRG3, and NDRG4, sharing 53–65% sequence similarity [50] The C-terminus of NDRG proteins contains residues, which are potential targets for kinases [50, 51]. Numerous studies have implicated NDRG1 as the key target of iron chelation with few implicating NDRG2 and NDRG3. NDRG1 expression has been linked to cell differentiation, adhesion, development and p53 dependent apoptosis [52–55]. Despite a strong relation between iron chelators, a link between NDRG1 and natural iron chelators has not been explored in the literature.

There is conflicting evidence on whether NDRG1 should be classified as a tumour suppressor or an oncogene. NDRG1 can display pleiotropy - performing different roles in different cell types and conditions [56]. Low levels of NDRG1 have been associated positive patient prognosis in oesophageal squamous cell carcinoma and neuroblastoma, yet poor patient prognosis in gastric cancer, breast cancer and hepatocellular carcinoma [57–60]. Pre-metastatic tumours are more likely to be hypoxic; this potentially explains higher levels of NDRG1 expression potentially through HIF-1α. Post translational modifications (PTM) could be another driver of pleiotropy. PTMs have been shown to influence NDRG1 cellular localisation [61]. Phospo-NDRG1 (Serine 330) localises within the nucleus, while Phospho-NDRG1 (Threonine 346) localises within the cytoplasm. PTEN gene silencing has the ability to increase Thr346 phosphorylation in prostate cancer cells, but not in hepatocellular carcinoma [61]. This further supports a pleiotropic mode of action. Treatment with DFO and Dp44mT both increase Thr346 and Ser330 phosphorylation [62]. Western blot analysis of DU145 cell lysates demonstrated two bands corresponding to NDRG1 one at 41kDa and one at 46kDa from DU145. The 41kDa band represented the truncated isoform of NDRG1, which is expressed in prostate cancer cell lines; PC3, DU145, LNCaP but not normal prostate cells PrEC [63]. Truncated NDRG1 has also been observed in PANC-1 and HT-29 cells. The truncated isoform demonstrates lowered nuclear localisation than the full-length isoform [61]. Localisation of proteins can have a major impact on classification as an oncogene of a tumour suppressor. Taken together, this suggests the truncated isoform may serve a role during carcinogenesis. Future studies should be sure to classify and distinguish between truncated and wild type NDRG1 isoforms.

### Uncontrolled cell growth and proliferation: Iron chelation attenuates cancer cell proliferation, enhancing signalling pathways

Uncontrolled proliferation is the most well recognised hallmark of cancer. During malignant transformation, cancer cells acquire changes in signalling pathways which amplify their proliferative potential. A plethora of evidence has linked iron chelation mediated NDRG1 upregulation to the inhibition of many oncogenic signalling pathways including STAT3, Wnt/ β-catenin, RAS and AKT/PI3K [62, 64–66]. Overexpression of NDRG1 - driven by iron chelators Dp44mT and DFO - in MIAPaCa-2 pancreatic cancer cell line lead to a downregulation of the oncogenic TGF-β and RAS pathways through an upregulation of SMAD4 (the pathway’s negative regulator). As a result, RAS downstream signalling molecules pERK, pSMAD2L are inhibited [64]. STAT3 is a transcription factor, which is constitutively active in tumours [67]. DFO, Dp44mT and DpC inhibit upstream kinases c-BCL and SRC, which promote STAT3 phosphorylation and subsequent dimerisation. As a result, STAT3 cannot promote the expression of its target genes: Bcl-2, cyclin D1, and c-myc [65].

NDRG1 can also suppress AKT and ERK signalling pathways. NDRG1 upregulation mediated through iron chelation has been shown to elevate PTEN expression levels in DU145 (prostate cancer cells), PrEC (normal prostate epithelial cells), but not PC3 (prostate cancer cells), which harbour a homozygous deletion in PTEN [62, 68]. While iron chelation did not increase the levels of AKT total protein it did increase the levels of pAKT (Ser473) in prostate cancer cells, whereas in Caco-2 cells there was a decrease in pAKT (Thr308) and no change in (Ser473) [62, 66]. The downstream effectors of pAKT – p-mTOR, S6K1, S6, 4E-BP1 and cyclin D1 - were not activated, despite the significant rise in pAKT [62, 66]. Both phosphorylation sites must be phosphorylated for pAKT to fulfil its role as a kinase [69]. Moreover, DFO stimulates REDD1 gene expression, which inhibits mTORC1 action [66]. Despite PC3 cells harbouring a deletion in PTEN, iron chelation still had an antiproliferative effect and still blocked AKT signalling, so hypothetically tumours with mutations in PTEN could still be treated with iron chelators. DFO and Dp44mT altered the ratio of p-SMAD2C and p-SMAD2L through decreasing the levels of oncogenic p-SMAD2L and p-SMAD2C levels remaining the same [62]. Levels of pERK1/2 dropped following iron chelation, suggesting pERK1/2 maybe responsible for the altered p-SMAD2L/p-SMAD2C ratio. NDRG1 overexpression mimicked the impact of iron chelation on AKT signalling, while NDRG1 silencing had the reverse effect, suggesting NDRG1 is responsible for the attenuation of AKT signalling. However, NDRG1 overexpression only slightly decreased the levels of pERK1/2 and p-SMAD2L so there may be another mechanism of signalling inhibition [62]. In contrast, treating triple negative breast cancer cells with DFO induces IL-6/PI3K/AKT signalling leading to an upregulation of iron uptake proteins Transferrin 1 and DMT1thus increasing iron uptake and supporting the pleiotropic function of NDRG1 [70]. It is possible that despite the capacity of NDRG1 to act as an oncogene, treating cells with iron chelators could return its tumour suppressive functions in certain conditions.

Wnt/β-catenin signalling represents another oncogenic signalling pathway attenuated by iron chelators. Deferasirox (DFX) inhibits Pyk2 phosphorylation without impacting total levels of Pyk2 protein, increasing β-catenin degradation through GSK-3β activation, thus impairing Wnt/β-catenin signalling [71]. Moreover, iron chelation by acyl hydrazones targeted β-catenin for ubiquitin mediated degradation even in cells with an abrogated destruction complex [72]. NDRG1 overexpression has been shown to increase β-catenin expression at the cell membrane and decrease β-catenin nuclear expression leading to lowered TCF/LEF signalling [73]. NDRG1 can interact with the Wnt receptor LRP6 in prostate cancer cells, inhibiting Wnt signalling cascade [74]. Cyclin D1 expression is a target gene of Wnt/ β-catenin signalling [75]. NDRG1 knockout lead to an increase in cyclin D1 levels [76].

It has been hypothesised that the ability of NDRG1 to block a range of oncogenic signalling pathways can be attributed to EGFR signalling as many of the pathways described previously are influenced by EGFR signalling. NDRG1 overexpression driven by Dp44mT and DpC in PANC-1 cells increases the half-life MI6 which facilitates EGFR degradation through lysosomal processing. This was dependent on PTEN expression [77]. Dp44mT can also prevent HER2 and HER3 activation leading to lower levels of EGFR/HER2 and EGF/HER3 heterodimer formation. Strikingly, Dp44mT has higher antiproliferative activity than the EGFR inhibitor erlotinib. The IC_50_ of erlotinib exceeds 80 μM in PANC-1, HT-29 and MIAPaCa-2 cells, whereas Dp44mT has an IC_50_ 0.02–0.04 μM [78]. When proliferative signalling exceeds a certain threshold, it can induce senescence [47]. VLX600 is an iron chelator which can target senescent and actively proliferating proving that cytotoxicity driven by iron chelation is not always dependent on cell cycle progression [38].

### Uncontrolled cell growth and proliferation: Iron chelation induces cell cycle arrest

The inhibition of cell proliferation pathways will have a domino effect across many cellular functions including cell cycle progression. Cells treated with iron chelators exhibit cell cycle arrest at two phases of the cell cycle: mid G1 phase and late G1/ early S phase [79, 80]. It has been predicted that an iron dependent checkpoint exists between G1 and S phase as cells depleted from iron will not enter S phase unless they had already reached S phase [79–81]. The mode of action of CDK1 is reliant on iron, which activates its kinase activities stimulating JAK/STAT3 signalling. Treatment with DFO inhibits CDK1 and its downstream signalling pathways [82]. Iron chelation can also elevate levels of cyclin E - a cyclin associated with mid G1 phase [83]. MDA MB 453 Breast cancer cells treated with mimosine have lowered levels of CDK4 and cyclin D – another CDK/cyclin complex which manifests during G1 phase [80].

Ribonucleotide reductase (RR) is an enzyme which provides cells with dNTPs - the building blocks of DNA. RR protein expression peaks during late G1 phase and early S phase to initiate the rate limiting step of DNA synthesis [84]. Iron is an enzyme cofactor for RR. When the binuclear iron reacts with oxygen a tyrosyl radical forms which is essential for RR enzyme activity [85]. The iron centre is labile and therefore requires constant replacing and as a result RR activity is dependent on the labile iron supply of the cell [85]. Iron chelators DFO, triapine and Dp44mT are capable of inhibiting RR, but through different mechanisms. DFO chelates the labile iron pool causing the disappearance of the tyrosyl radical and loss of RR enzyme activity [86]. Dp44mT inhibits RR activity through its impact on thiol antioxidant systems: thioredoxin, glutaredoxin and glutathione, which play a key role in maintaining RR protein double bonds [87]. Triapine has been suggested to bind directly to a binding pocket on the surface of the R2 subunit of RR freeing the diferric centre in mouse models [22]. Nevertheless, the net result is RR activity inhibition and cell cycle arrest.

### Evading growth suppression: Iron chelation reactivates tumour suppressor genes

Tumour suppressor genes safeguard normal cells from uncontrolled growth and proliferation. During malignant transformation many tumour suppressor genes are mutated or switched off, therefore it is useful for new cancer therapies to be able to return of tumour suppressive signalling. NDRG1 is a potential tumour suppressor gene that has been implicated in iron chelator mediated cytotoxicity. NDRG1 gene expression is upregulated following treatment with DFO, 311 and Dp44mT and is attenuated when cells were supplemented with iron salts [88]. This suggests NDRG1 expression is stimulated by iron chelation rather than ROS induction. When NDRG1 is knocked out, cells become less sensitive to iron chelation, suggesting a crucial role for NDRG1 in the mode of iron chelator action. Interestingly, overexpression of NDRG1 combined with iron chelation treatment does not further sensitise cells to iron chelators [89]. There are several ways iron chelation could be inducing NDRG1 gene expression. Hypoxia-inducible factor 1α (HIF-1α) can promote NDRG1 gene expression through a Hif response element (HRE) sequence in the promoter region of NDRG1, however HIF-1α is not essential for NDRG1 gene expression [90]. In HIF-1α knockout (HIF-1α-/-) murine embryo fibroblasts (MEFs), which lack wild type HIF-1α protein expression iron chelation could still induce NDRG1 expression under normoxic conditions but not hypoxic conditions [88]. SP1, CEBPα, YB-1, Smad7 and p53 have also been proposed as transcriptional factors which upregulate NDRG1 gene expression [53, 88]. NDRG1 expression is repressed by oncogenic C-MYC and N-MYC [91]. NDRG1 was upregulated in p53 wild type MCF7 cells and p53 deficient H1299 to same extent in response to DFO and 311, suggesting this is independent of p53 expression [88].

Cell cycle progression is driven by components which are perceived as oncogenic (cyclins and cyclin dependent kinases) and inhibited by tumour suppressors (cyclin dependent kinase inhibitors). Iron chelation can inhibit oncogenic cell cycle drivers (e.g. cyclin D, CDK4), and simultaneously activate the tumour suppressive CDK inhibitors driving cell cycle arrest. Interestingly, iron chelation can modulate p21 levels and nuclear localisation independently of p53 status. Dp44mT can downregulate p21 in MCF7 (Breast cancer) cells, upregulate p21 in SK-MEL-28 (Melanoma) and CFPAC-1 (Pancreatic ductal adenocarcinoma) cells and has no effect on p21 in LNCaP (Prostate cancer) and SK-N-MC (Neuroepithelioma cell line derived from a supra orbital brain tumor) cells [92]. This is thought to be through MDM2. Iron chelation by DFO increases MDM2 gene expression, this is reversed by iron overload, as MDM2 expression is regulated by Iron Regulatory Protein 2 (IRP2) [93–95]. DFO can elevate the expression of p27 in serum-stimulated 3T3 cells inhibiting CDK2/cyclin E activity and preventing S phase entry [81]. DFO inhibits src kinase mediated phosphorylation and degradation of p27 [96]. Iron chelation increases levels of tumour suppressor gene PTEN and SMAD4 (detailed above) [64]. In addition to this, PTEN can upregulate NDRG1 gene expression suggesting a positive feedback loop [97].

### Resisting programmed cell death: Iron chelation induces apoptotic signalling and the unfolded protein response

Normal cells have built-in mechanisms to prevent replication across damage thus passing on mutation. Among these mechanisms is apoptosis, which plays a vital role in growth and development of multicellular organisms and provides protection against carcinogenesis. Cancer cells develop mutations which prevent normal apoptotic signalling. Iron chelation has been shown to induce apoptosis *in vitro* and *in vivo*. This is accompanied by an increase in the levels of pro-apoptotic proteins such as BAX, caspase 8 caspase 9 and caspase 3 and a drop in anti-apoptotic proteins such as Bcl-2 as well as a release of cytochrome C [34, 98–103]. The dual involvement of caspase 8 and 9 suggests a death receptor and mitochondrial apoptotic cascade. Iron chelation by DFO induces an increase in p53 protein levels but not the mRNA levels indicating post-transcriptional regulation. The iron chelators 311 and Dp44mT have also been observed to increase p53 protein expression [99, 104, 105]. It has been established that an excess of iron leads to a downregulation of p53. Iron polyporphyrin heme triggers p53 degradation and blocks p53-DNA interactions [106]. However, iron chelator Dp44mT can induce p53 independent cell death in cell lines with non-functional p53 mutations such as PC-3 cells [33]. The effectiveness of iron chelation is therefore not dependent on p53.

Iron chelators DFO, DFX and Dp44mT induce ER stress through the four main modules of the unfolded protein response: protein kinase RNA-like endoplasmic reticulum kinase (PERK), inositol-requiring enzyme 1 (IRE1), activating transcription factor 6 (ATF6) and CaMKII. As a result of iron chelation mediated ER stress, JNK signalling is activated; a crucial signalling pathway for ROS induced cell death, and this initiates apoptotic signalling, therefore iron chelation mediated apoptosis is induced through ER stress [103, 107]. Interestingly, non-ROS inducing iron chelator DFO can stimulate JNK signalling [108].

The MAPK subfamilies p38, and JNK have been implicated in iron chelation induced death, however studies have not been consistent in assigning which signalling module is primarily responsible. The p38 MAPK was shown to be the primary inducer of apoptosis in HL-60 cells (human promyeloid leukemic cells), as p38 activation was observed prior to the early apoptotic traits and blocking p38 activity prevented the apoptotic cascade. Only low levels of JNK activation at the late stages of apoptosis was observed in HL-60 cells, whereas in gastric cancer cells AGS and SNU638 there was a rise in JNK phosphorylation and inhibiting JNK lead to a halt in iron chelation mediated cell death [101, 103].

In addition to this, iron chelation has been shown to induce autophagy. Interestingly, DFO and Dp44mT can drive LC3-I to LC3-II transformation - a marker of the autophagosome - through the PERK/eIF2α axis but NDRG1 overexpression represses pro-survival autophagy [109].

DFO and Dp44mT induced severe phenotypic changes in MDA MB 231 and MDA MB 157 breast cancer cell lines that closely resembled non-apoptotic non-autophagic cell death methuosis [110]. This was thought to be a survival adaptation allowing cells to accumulate nutrients extracellularly. The cells displayed lipid droplet accumulation, mitochondrial defects, a halt in protein translation and eventual cell death. These phenomena were undetected in any other cell lines [110]. Potentially different signalling pathways could trigger cell death in different cell lines. Another intriguing cellular response was silibinin induced mitophagy. Breast cancer cells MDA MB 231, MCF7 cells treated with silibinin suffered from mitochondrial fission, which lead up to mitophagy and apoptosis [111]. Ferroptosis is a form of cell death driven by iron accumulation, and ROS induction. Events observed during ferroptosis include lipid peroxidation, GPX4 depletion and cell rounding. As expected DFO can inhibit the onset of ferroptosis, but cannot reverse ongoing ferroptosis [112].

### Enabling replicative immortality: Iron chelation inhibits stemness and Wnt/β-catenin signalling

In a normal cell population, there is a limit to the number of achievable cell doublings before cells reach their natural fate – senescence. Cancer cells lose this attribute to enable indefinite replication often showcasing stem cell like features. Enhanced telomerase activity is a driver of cancer’s immortality. Wnt/β-catenin signalling has been shown to directly induce telomerase transcription [113]. As mentioned previously iron chelation downregulates Wnt/β-catenin signalling and prevents nuclear translocation of β-catenin abrogating any changes in target gene expression. Iron chelation could potentially block replicative immortality, but this has not been directly investigated. Iron depletion by DFX hindered the expression of stemness markers Nanog, Oct3/4, Sox2, Klf4, and c-Myc and inhibited spherogenicity in colorectal and lung cancer stem cell models *in vivo* and *in vitro.* In addition to this, iron chelators DFO and DFX are cytotoxic to cancer stem cells which are resistant to classical chemotherapeutic drugs [114, 115]. Epigallocatechin gallate is also capable of inhibiting the expression of stem cell markers (CD)44, CD133, Oct4, ALDH1A1 and Nanog, through inhibiting sonic hedgehog signalling [116].

### Sustained angiogenesis: Iron chelators demonstrate pro and anti-angiogenic capabilities

Angiogenesis is the creation of new blood vessels which sprout from pre-existing vessels improving access to nutrients and oxygen as well as increasing the likelihood of metastasis [47, 117]. Studies have been unclear and inconsistent regarding whether iron chelation can inhibit or stimulate angiogenesis. Iron chelation can inhibit PI3K signalling which is a proangiogenic pathway [64, 118]. NDRG1 upregulation has also been shown to downregulate MMP2 and MMP9 - key mediators of angiogenesis [119, 120]. On the other hand, DFO and Dp44mT upregulate VEGF expression through HIF- α, as hypoxia is a driver of angiogenesis [35, 117]. This is concerning as iron chelation has been proven to induce hypoxia [121]. Interestingly, VEGF gene expression is driven by HIF-α and STAT3 binding to the promoter region simultaneously [122]. As mentioned previously iron chelators prevent STAT3 activation and blocking STAT3 activity has been shown to inhibit VEGF gene expression [65, 123]. Iron chelation has been shown to induce macular edema *in vivo* [124]. This suggests iron chelation may induce angiogenesis. The reverse has been observed with natural iron chelators with silibinin, quercetin and epigallocatechin gallate decreasing the levels of angiogenesis associated proteins VEGF, MMP2, MMP9, and HIF-1α [125–128]. In fact, quercetin has been shown to inhibit angiogenic protein expression through STAT3 signalling inhibition [127]. The impact of iron chelation on angiogenesis *in vivo* has not been investigated. Iron chelators could potentially be combined with an angiogenesis inhibitor to prevent angiogenesis promotion induced by iron chelation.

### Activating migration and metastasis Iron chelation prevents the initiation of metastasis through EMT and ROCK/MLC2 and NF-kB inhibition

Epithelial-mesenchymal transition (EMT) is a morphological change occurring during embryogenesis and wound healing. Cancer cells exploit the EMT to drive their migration and invasion. During the EMT cells undergo morphological and biochemical changes transforming them from an epithelial phenotype to a mesenchymal phenotype. TGF-β signalling promotes EMT and migration during the later stages of carcinogenesis [129]. When HT29 (colorectal cancer) or DU145 (prostate cancer) cells are treated with TGF-β, they undergo EMT which is characterised by a more spindle-like cell morphology, the presence of mesenchymal marker vimentin and a decrease in epithelial markers β-catenin and E-cadherin at the cell surface membrane. Combining TGF-β with iron chelators prevents TGF-β mediated EMT as displayed by a retained epithelial morphology and markers [76]. NDGR1 has been observed near the adhernes junctions and desomosomes in the cytoplasm so it possible that NDRG1 plays a role in cell adhesion through the formation of the E-cadherin/ β-catenin complex [56]. Another way the EMT is blocked is through the attenuation of NF-kB signalling. During tumorigenic conditions, the EMT is driven through TNF-α mediated LYRIC expression, which upregulates NF-kB signalling and in turn vimentin gene expression is induced. Treating cells with Dp44mT and DpC prevents this signalling cascade from occurring through NDRG1 upregulation. NDRG1 overexpression inhibits NF-ĸB signalling through reducing NEMO expression, preventing activation of Iĸĸα/β and blocking nuclear localisation of p65 [130]. During the EMT E-cadherin is downregulated by the transcriptional factors SLUG, SNAIL, TWIST and ZEB2. Iron chelation driven NDRG1 expression downregulates SLUG and SNAIL, whereas NDRG1 knockout has the reverse effect [76]. SMAD molecules are modulators of TGF-β signalling. Knockout of NDRG1 increased SMAD2, pSMAD3, and SMAD4 and overexpression lead to decreased SMAD2 and pSMAD3. These could mean NDRG1 regulation of TGF-β signalling could be through SMAD molecules [62, 76]. On the other hand, treating aggressive breast cancer cells, MDA MB 231 with DFO caused them to accumulate iron and acquire mesenchymal markers through activating TNF-alpha, NF-ĸB and TGF-β signalling. DFO was still effective in blocking EMT in non-aggressive MCF7 breast cancer cells [131].

Dp44mT and DFO have been shown to inhibit cell migration through NDRG1 mediated suppression of the Rho associated coiled-coil-forming protein kinase 1/myosin light chain 2 (ROCK1/pMLC2) pathway. ROCK1 phosphorylates MLC2 and this induces cell migration through actomyosin contractility [132, 133]. MLC2 drives cell motility through linking anti-parallel actin filaments causing sliding and forming stress fibres [132, 134]. Metastasis signalling is a trigger of stress fibre formation, therefore inhibiting MLC2 activity may inhibit metastasis [135]. Iron chelation treatment in HT29, HCT116 and DU145 cells decreases the levels of ROCK1 and phosphorylated MLC2 with no change in basal MLC2. Moreover, the levels of F-actin were also decreased making stress fibres less likely to form and attenuating cell motility and metastasis [136].

### Genomic instability and mutation: Iron chelation exploits and mimics genomic instability

Many cancers have defective DNA damage repair, giving rise to mutants that will survive new selection pressures. As a result, many current cancer therapies exploit the genomic instability of cancer cells by causing irreparable DNA damage or inhibiting DNA damage repair eventually triggering cell death. ROS inducing iron chelators such as Dp44mT can induce double strand breaks [137–139]. Interestingly, iron chelators can not only induce DNA damage, but they can also inhibit homologous recombination repair DNA damage repair. Dp44mT and Triapine have been shown to inhibit ribonucleotide reductase preventing the production of the dNTP precursors of DNA damage repair [137]. Moreover, triapine can sensitise BRCA wildtype and PARP resistant epithelial ovarian cancer cells and xenograft mice to PARP inhibitor olaparib [140]. This is beneficial as reversal of BRCA mutations and a return of homologous recombination repair functioning have been observed in ovarian cancer patients [140–143]. Triapine combined with olaparib prevents homologous recombination repair of double strand breaks in wild type BRCA ovarian cancer cells through preventing RAD51 and BRCA1 foci formation as well as preventing BRCA1 from associating with the MRN complex and attenuation of CtIP phosphorylation [144]. In summary triapine blocks all olaparib driven homologous recombination repair in BRCA wildtype ovarian cancer cells, mimicking BRCAness [144, 145]. Cyclin D1 plays a role in mediating homologous recombination repair. Ionising radiation induces a cyclin D1/RAD51 interaction and inhibition of cyclin D1 expression sensitises cancer cells to ionising radiation [146]. Triapine could potentially be inhibiting HR through blocking cyclin D1 gene expression.

### Metabolic reprogramming: Novel iron chelator VLX600 targets oxidative phosphorylation

Cancer cells undergo a metabolic transformation known as the Warburg effect, which shifts their source of energy from oxidative phosphorylation to glycolysis. This is another trait which is exploited by iron chelators. VLX600 diminishes the ability of MCF7 and HCT116 cells to undergo oxidative phosphorylation [38]. Triapine, DFO and CPX can inhibit oxidative phosphorylation but to a lesser extent. VLX600 reduces hypoxia which suggests it has lowered cell oxygen consumption. VLX600 also reduces cytochrome oxidase (complex IV) activity - the rate limiting step of oxidative phosphorylation [38]. The cells in the deep layers of a tumour are vulnerable to even the slightest decrease in oxidative phosphorylation, so theoretically they would be highly sensitive to VLX600 [38, 147, 148]. DFO can inhibit aerobic glycolysis and oxidative phosphorylation through ERK1/2 inhibition and reduce the gene expression of the 13 mitochondrial complex components [149].

Iron chelators can also impact iron metabolism. DFO targets ferritin for degradation through autophagy, whereas DFX and deferiprone target ferritin for proteasomal degradation [150]. Quercetin not only potently forms complexes with iron but can also induce cellular iron deficient behaviour such as induction of transferrin receptor-1 and iron regulatory protein-2 expression and lowered ferritin expression. Additionally, quercetin can modulate iron metabolism gene expression in rats decreasing expression of DMT1, Dcytb, FPN, and hepcidin. This decreased the level of iron absorption [151]. Dp44mT induced transferrin expression in DMS-53 xenograft mice, which could potentially lead to iron accumulation [35]. Although counterintuitive, iron chelation of MDA MB 231 cells by DFO lead to iron accumulation through an increase in TfR1 and DMT1 expression. This is thought to be a survival adaptation [152].

## CONCLUSIONS AND FUTURE WORK

Iron chelation has proven to be successful on a range of different tumour types *in vitro* and *in vivo* as iron addiction is a universal cancer trait. Even cancers which are notoriously hard to treat due to resistance have been subdued by iron chelation such as androgen insensitive prostate cancer and cancer stem cells or cells with loss of p53 and PTEN function.

Based on the data presented in this review iron chelators could potentially reverse many of the key hallmarks of cancer. Stripping the cells of iron impacts many cellular targets with some targets still undiscovered. NDRG1 has been proven to be the common link between the ability of iron chelators to reverse many of the hallmarks of cancer as overexpression of NDRG1 mimics the impact of iron chelation on several signalling pathways. Problems may arise from treating aggressive breast cancer cells with iron chelators. Aggressive breast cancer cells have demonstrated a unique reaction to iron chelators, including the accumulation of iron, activation of oncogenic signalling pathways and a methuosis-like death. Another potential issue is the ability of iron chelators to induce autophagy, which can function as a pro-survival response and a tumour suppressor response in cancer cells. There are still many unanswered questions about the mechanism of action of iron chelators. A consensus must be reached on the impact of iron chelation on angiogenesis through *in vivo* studies. As STAT3 is essential for VEGF gene expression and iron chelation attenuates STAT3 dimerisation and nuclear localisation. Studies must confirm if STAT3 is still capable of inducing VEGF gene expression in cells treated with iron chelators such as Dp44mT, as this has been confirmed with epigallocatechin gallate. If more evidence is found linking iron chelation and angiogenesis, iron chelation could potentially be combined with an angiogenesis inhibitor to prevent angiogenesis promotion. Any future studies on the impact of NDRG1 on patient prognosis or tumorigenesis must differentiate between the cleaved and full isoforms as well as the phosphorylation isoforms. The cleaved isoform is only present in cancer cells and could potentially be oncogenic. Although many mechanistic studies have been undertaken iron chelators, the complexity of cell signalling remains a hurdle preventing the discovery of all cellular targets of iron chelation. A potential way of discovering new targets is combining iron chelators with well-characterised cancer therapeutics. Triapine was discovered to inhibit homologous recombination repair as a result of combinatorial studies. We propose a combinatorial study of iron chelators with immune checkpoint inhibitors as they have shown success in clinic and could uncover more mechanisms of action. The full impact of iron chelators on the two remaining hallmarks of cancer inflammation and immune evasion must be established. Moreover, iron chelation has multiple targets within a cancer cell, so the question lies whether the ideal cancer therapy is overarching or specific.

### Note

References [153–218] are present in the main article, whereas their citations are called out in the Supplementary Materials.

## SUPPLEMENTARY MATERIALS






