# Supplementary material for: Reversing oncogenic transformation with iron chelation
Source: Oncotarget. 2021 Jan 19;12(2):106–24. doi: 10.18632/oncotarget.27866 (PMC7825639; doi:10.18632/oncotarget.27866)
Supplement: Supplementary file 1 [file oncotarget-12-106-s001.pdf]

Reversing oncogenic transformation with iron chelation

SUPPLEMENTARY MATERIALS

Supplementary Table 1: The mechanism behind iron chelation. See Supplementary Table 1

Supplementary Table 2: Iron chelators response in clinical trials. See Supplementary Table 2
