# Supplementary material for: Reversing oncogenic transformation with iron chelation
Source: Oncotarget. 2021 Jan 19;12(2):106–24. doi: 10.18632/oncotarget.27866 (PMC7825639; doi:10.18632/oncotarget.27866)
Supplement: Supplementary file 2 [file oncotarget-12-106-s002.docx]

**Supplementary Table 1: The mechanism behind iron chelation**

| Iron chelator | Uncontrolled cell growth and proliferation | Evading growth suppression | Resisting programmed cell death | Enabling replicative immortality | Sustained angiogenesis | Activating migration and metastasis | Genomic instability and mutation | Metabolic reprogramming |
| --- | --- | --- | --- | --- | --- | --- | --- | --- |
| DFO | STAT3 [65], Wnt/ β-catenin [76], RAS [62], AKT/PI3K [62],  CDK1 [82]  CDK4 [80]  Cyclin D [80]  cyclin D1, D2, and D3 and Cdk2 [153]  Ribonucleotide reductase inhibition [86] | NDRG1 [65]  PTEN [64]  SMAD4 [64]  p27 [81]  p21  [92] | Apoptosis [154]  ER stress [103]  Autophagy [109],  Methuosis [110] | Wnt/ β-catenin [76]  Stemness [115] | PI3K [62]  VEGF [35] | TGF- β [76]  ROCK/MLC2 [136] | Ribonucleotide reductase [86] | Oxidative phosphorylation,  Glycolysis [149]  Iron metabolism [152] |
| DFX | AKT [155], ERK [155]  Wnt/ β-catenin [71]  Ribonucleotide inhibition [156] | p53 [157] | Apoptosis [154]  ER stress [103] | Wnt/ β-catenin [71]  Stemness [114] | VEGF [158]  HIF-1alpha [159] | NF-ĸb [155] | Ribonucleotide inhibition [156] |  |
| Mimosine | ERK [160]  CDK4 [80]  Cyclin D [80]  Cyclin E [161] | p21 [161] | Apoptosis [160] |  |  |  |  |  |
| L-mimosine derivative compound 22 |  |  | Apoptosis [162] |  |  |  |  |  |
| Triapine | Ribonucleotide reductase inhibition [22]  cyclin D1, D2, and D3 and Cdk2 [153] |  | Apoptosis [163] |  |  |  | Ribonucleotide inhibition [22]  Homologous recombination inhibition [140] |  |
| Dp44mT | STAT3 [65] Wnt/ β-catenin [76] RAS [62], AKT/PI3K [62], EGFR signalling [71]  Ribonucleotide reductase inhibition [87] | NDRG1 [65]  PTEN [64]  SMAD4 [64]  p21 [92] | Apoptosis [33]  ER stress [103, 107]  Autophagy [109]  Methuosis [110] | Wnt/ β-catenin  [76] | PI3K [62] VEGF  [35] | TGF-beta [76]  NF-ĸb [130]  ROCK/MLC2 [136] | Double strand breaks [137]  Ribonucleotide reductase [87] |  |
| DpC | EGFR signalling [71] |  | Apoptosis [164] |  |  | NF- ĸb [130] |  |  |
| 311 | Ribonucleotide reductase [165]  cyclin D1, D2, and D3 and Cdk2 [153] | p21[105] | p53 [166]  p21 [92] |  |  |  | Ribonucleotide reductase [165] |  |
| VLX600 |  |  | Autophagy [167] |  |  |  | Double strand breaks [168] | Oxidative phosphorylation [38] |
| 8-Hydroxyquinoline |  |  |  | Stemness [169] |  | NF- ĸb [169] | Double strand breaks [170] |  |
| Tachypyridine |  | p53 [171] | Apoptosis [171] |  |  |  |  |  |
| Silibinin | ERK/AKT [128]  JAK/STAT3 [172]  mTOR [173]  CDK4, CDK2,  cyclins D1, D3 E [174] | p18, p21, p27 [174] | Apoptosis [172, 175]  Autophagy  [176]  Mitophagy [111] | Stemness [177] | MMP2, MMP9 [128] | E-cadherin, Slug, Snail, β-catenin [178] |  | Lipid metabolism  [179] |
| Quercetin | AKT/AMPK/mTOR [180] Wnt/ β-catenin [181] | p21, p53, p27 [182] | Apoptosis  [183] | Stemness [184]  Wnt/ β-catenin [181] | HIF-1α, VEGF, MMP2, and MMP9 [127] | EMT [185] |  | TfR1, Ferritin, IRP2 [186] |
| Catechin gallate | Cyclin D1, cyclin D3, cyclin B1, CDK4, CDK6 and cdc2 [187] | p21, p27, p15 [187]  p53 [188] | Apoptosis [189] |  |  |  |  |  |
| Epicatechin gallate | Cyclin D1, cyclin D3, cyclin B1, CDK4, CDK6 and cdc2 [187] | p21, p27, p15, Rb [187] p53 [188] |  |  |  |  |  |  |
| Epigallocatechin gallate | PI3K/AKT [190], Wnt/ β-catenin [191] Sonic hedgehog [116]  STAT3 [126]  TGF- β [192]  cyclin D1, cyclin E, cdk2, cdk4, and cdk6 [193]  EGFR, HER2 [194] | PTEN [190]  p21 [195]  p27, p16, p18 [193]  p53 [196] | Apoptosis [190] | Wnt/ β-catenin [191]  Sonic hedgehog [116] | MMP2, MMP9 [125]  STAT3, VEGF [126] | EMT [192] |  |  |
