# Supplementary material for: Reversing oncogenic transformation with iron chelation
Source: Oncotarget. 2021 Jan 19;12(2):106–24. doi: 10.18632/oncotarget.27866 (PMC7825639; doi:10.18632/oncotarget.27866)
Supplement: Supplementary file 3 [file oncotarget-12-106-s003.docx]

**Supplementary Table 2: Iron chelators response in clinical trials**

| Treatment | Number of patients | Patient response | Reference |
| --- | --- | --- | --- |
| DFO | 5 children with neuroblastoma | 1 patient had complete response lasting for 4 months | [13] |
| DFO | 10 children with recurrent neuroblastoma | No partial or complete response | [14] |
| DFO | 9 patients with neuroblastoma | 7 patients experienced 50% decrease in bone marrow infiltration. One patient experienced complete clearance. Another patient had a 48% decrease in tumour growth | [197] |
| DFO, cyclophosphamide, etoposide, carboplatin, and  thiotepa (D-ECaT) | 13 patients with Stages III or IV of neuroblastoma | 1 patient experienced very good partial response * 3 experienced partial response | [15] |
| DFO, cyclophosphamide, etoposide, carboplatin and thiotepa | 65 patients with Stage III or IV neuroblastoma | 24 complete responses, 5 very good responses, 21 partial response,  3 minor responses, and 4 progressive disease | [12] |
| Triapine | 27 patients with advanced cancer | 8 patients experienced disease stabilisation. | **[27]** |
| Triapine and Gemcitabine | 26 patients with advanced pancreatic cancer | 11 patients experienced disease stabilisation- lasting more than 6 months for 5 of those patients | [198] |
| Triapine | 20 patients with advanced solid tumours | 5 patients experienced disease stabilisation | **[199]** |
| Triapine and Gemcitabine | 33 patients with Advanced biliary tract cancer | 3 out of 23 patients with normal liver function experienced objective response | **[200]** |
| Triapine and cytarabine | 25 patients with relapsed or refractory myeloid leukaemia | 2 complete and 1 partial response. 1 elderly patient experienced haematologic improvement | **[201]** |
| Triapine and Gemcitabine | 22 patients with advanced cancer | 1 complete response and two partial responses. One patient with large liver metastasis experienced prolonged disease stabilisation. | **[202]** |
| Triapine | 21 patients with advanced cancer | Disease stabilisation in 4 patients | **[203]** |
| Triapine, cisplatin and pelvic radiation | 10 patients with locally advanced cervical cancer | 10 patients experienced prolonged complete response | **[204]** |
| Triapine | 15 advanced adenocarcinoma of the pancreas | No response | [25] |
| Triapine and fludarabine | 37 patients with aggressive myeloproliferative neoplasms | 49% overall response. 24% complete remission | **[24]** |
| Triapine | 30 patients with recurrent or metastatic head and neck squamous cell carcinoma | 5.9% overall response. 3.9 months to disease progression | [205] |
| Triapine | 24 patients with refractory leukaemia | No objective response  >50% reduction in white blood cells 70% of patients  Elimination of marrow leukemic blasts was observed in two patients with AML accompanied by aplasia of several weeks, and one patient with AML achieved a partial remission. | **[206]** |
| Triapine and Fludarabine | 33 patients with Refractory Acute Leukaemia and Aggressive Myeloproliferative Disorders | Complete + partial response of 21% for patients on schedule A (Triapine 105 mg/m2 /day for 4 hours followed by daily fludarabine treatment for 5 days) | **[207]** |
| Triapine and Gemcitabine | 12 Advanced Non-Small-Cell Lung Cancer patients | No objective response. 4 patients had disease stabilisation. Time to progression 3 months | **[208]** |
| Triapine and Cytarabine | 33 patients with Acute Leukaemia or Myelodysplastic Syndrome | Complete response in 14% of patients | **[209]** |
| Triapine and Gemcitabine | 15 patients with Advanced Non-Small Cell Lung Cancer | No objective responses. 20% experienced stable disease. | **[26]** |
| Triapine | 25 adults with advanced leukemias | No complete or partial response. >50% white blood cell count in 76% of patients | **[210]** |
| Triapine and gemcitabine | 36 patients with Advanced Solid Tumours | 1 patient had a partial response and 15 had stable disease of 30 evaluable patients. | **[211]** |
| Triapine and doxorubicin | 20 patients with advanced solid tumours | No objective response | **[212]** |
| Triapine | 32 patients with advanced solid tumours | No objective response | **[213]** |
| Triapine, cisplatin and radiotherapy | 24 patients with bulky IB-IIIB cervical cancer | 3 year relapse rate 4%, disease free survival 80%, and 82% overall survival | **[29]** |
| Triapine | 19 patients with metastatic renal cell carcinoma | Study discontinued | **[214]** |
| Triapine and cisplatin | 10 patients with advanced-stage solid tumor malignancies. | No objective response. 5 patients experienced disease stabilisation | **[30]** |
| VLX600 | 19 patients with refractory advanced solid tumours | No objective response. 6 patients had stable disease. | **[39]** |
| Silybin-phytosome | 12 patients with localised prostate cancer | Low silibinin tissue penetrance | **[215]** |
| Silybin-phytosome | 13 prostate cancer patients | No objective PSA response | **[216]** |
| Silybin phosphatidylcholine | Patients with advanced hepatocellular carcinoma | Could not determine maximum tolerated dose | **[217]** |
| Polyphenon E (Green tea catechin gallate compounds) | 26 prostate cancer patients about to undertake a prostatectomy | Lowered levels of PSA, HGF, and VEGF in patient serum | **[218]** |
